# Actual Ligation Frequencies in the Chromosome Conformation Capture Procedure

**DOI:** 10.1371/journal.pone.0060403

**Published:** 2013-03-26

**Authors:** Alexey A. Gavrilov, Arkadiy K. Golov, Sergey V. Razin

**Affiliations:** 1 Institute of Gene Biology of the Russian Academy of Sciences, Moscow, Russia; 2 Faculty of Biology, M.V. Lomonosov Moscow State University, Moscow, Russia; 3 University of Oslo, Center for Medical Studies in Russia, Moscow, Russia; 4 LIA 1066 French-Russian Joint Cancer Research Laboratory, Villejuif, France–Moscow, Russia; The National Institute of Diabetes and Digestive and Kidney Diseases, United States of America

## Abstract

Chromosome conformation capture (3C) and derivative experimental procedures are used to estimate the spatial proximity between different genomic elements, thus providing information about the 3D organization of genomic domains and whole genomes within the nucleus. All C-methods are based on the proximity ligation–the preferential ligation of joined DNA fragments obtained upon restriction enzyme digestion of in vivo cross-linked chromatin. Here, using the mouse beta-globin genes in erythroid cells as a model, we estimated the actual frequencies of ligation between the fragments bearing the promoter of the major beta-globin gene and its distant enhancers and showed that the number of ligation products produced does not exceed 1% of all fragments subjected to the ligation. Although this low yield of 3C ligation products may be explained entirely by technical issues, it may as well reflect a low frequency of interaction between DNA regulatory elements in vivo.

## Introduction

The current model of the functionally dependent architecture of interphase chromosomes is based, to a large extent, on the results obtained using the chromosome conformation capture (3C) technique and derivative experimental approaches (reviewed in [1]). The main principle underlying all C-methods is that the chromosome spatial configuration is fixed in living cells by formaldehyde, which “freezes” contacts between genomic elements. The resulting meshwork of cross-linked chromatin fibers is subjected to cleavage with restriction enzyme(s), followed by DNA ligation. The restriction fragments that are held in close spatial proximity due to the cross-linkages between DNA-bound proteins have an increased likelihood of meeting each other and thus becoming cross-ligated compared to fragments located far from each other in the nuclear space. Therefore, the ligation frequency of any two restriction fragments can be used to measure the relative spatial proximity of these fragments in the nuclear space [2], [3].

In a conventional 3C experiment, ligation frequencies are determined by qPCR with amplicons spanning ligation junctions of interest and expressed in units reflecting the relative amounts of the ligation products [4], [5]. Based on the analysis of relative frequencies of interaction of an arbitrary chosen anchor fragment with a set of fragments located at different distances from the anchor (along the DNA chain) one can find the fragments that are likely to reside in close spatial proximity to the anchor [2], [3]. As all biochemical protocols, the 3C cannot provide for estimating the proportion of cells in which two particular DNA sequences interact, and can only be used to analyzing the average interaction pattern for a given cell population [4], [6]. The portion of cells (chromosomes) in which the locus under study has a linear configuration cannot be estimated. If in a given cell population in some cells the locus under study has a looped and in the others–a linear configuration, the profiles of 3C signals (3C curves) will be very similar regardless the ratio of loci with looped and linear configuration. In order to get more insights into the quantitative aspects of the 3C protocol, we have determined absolute yield of ligation products in the 3C analysis of the mouse beta-globin gene locus in erythroid cells. We have measured the input quantity of DNA fragments taken in the 3C ligation reaction and the output quantity of ligation products and have found that the yield of ligation products does not exceed 1% for fragments that are assumed to be involved in direct spatial interactions.

## Results

For this study, we selected the mouse beta-globin locus, the spatial organization of which has been characterized extensively using the conventional 3C procedure [2], [7]–[9]. Previous studies revealed erythroid-specific interactions between the promoters of the major and minor beta-globin genes (*Hbb-b1* and *Hbb-b2*, respectively) and several enhancer sites, particularly DNAse I hypersensitive sites (HS) 1, 4 and 5 of the locus control region (LCR) and the -62/-60 HS sites. All these elements are suggested to interact with each other to form an active chromatin hub that is essential for beta-globin gene transcription [2].

**Figure 1:**
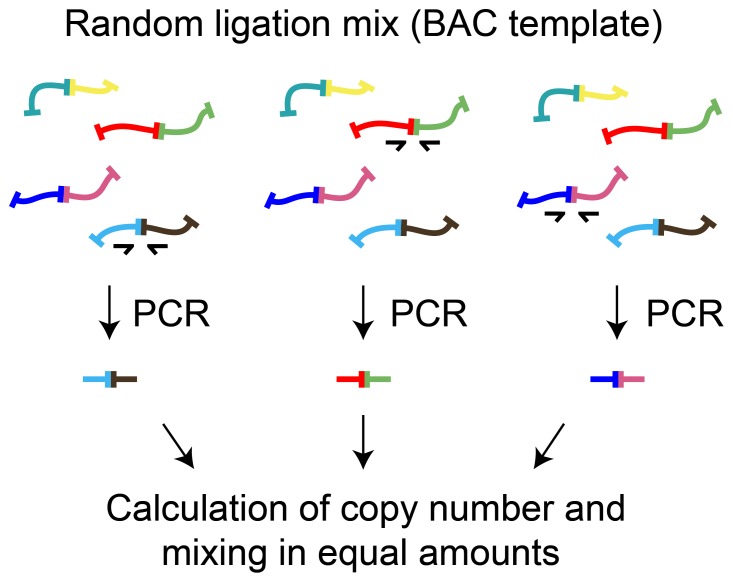
Preparation of the random ligation standard with DNA fragments of known copy number. A BAC random ligation template was amplified with 3C primers. PCR products were purified and, after their copy number was determined, mixed in equimolar amounts.

We reproduced the 3C experiments reported by Tolhius et al. (2002) using the restriction enzyme *Hind*III (for details, see the Materials and Methods section) and analyzed the frequency of ligation of the fragment containing the *Hbb-b1* promoter with several selected fragments of the locus (fragments “a-f”, see below). In a conventional 3C experiment, PCR signals from different pairs of primers are normalized to the signals obtained with the random ligation mix–a control template prepared by the ligation of equimolar amounts of the DNA fragments of interest (or their ends), which is assumed to contain equal amounts of all ligation products [4]–[6]. Using this standard one can only estimate the relative amounts of ligation products as the copy number of ligation products in the random ligation mix is not known. To determine the absolute yield of 3C ligation products, we prepared a standard equimolar mix of synthesized DNA fragments comprising the exact ligation products with known copy numbers of each fragment, as had been proposed by Comet et al. [10]. More precisely, the mix was composed of PCR-amplified DNA fragments covering ligation junctions of interest. A random ligation mix (the same as that used as a standard in 3C experiments; see the Materials and Methods section for details) was used as an amplification template, and the primers used were the same as those used for the subsequent analysis of ligation frequencies. The products of amplification were separated by gel electrophoresis and purified. After the determination of the DNA concentration in each sample, aliquots containing equal amounts of fragments were mixed (Figure 1). The obtained template was used to prepare a series of standard dilutions with a known copy number of target DNA sequences; these standards were amplified in parallel with the 3C template.

**Figure 2:**
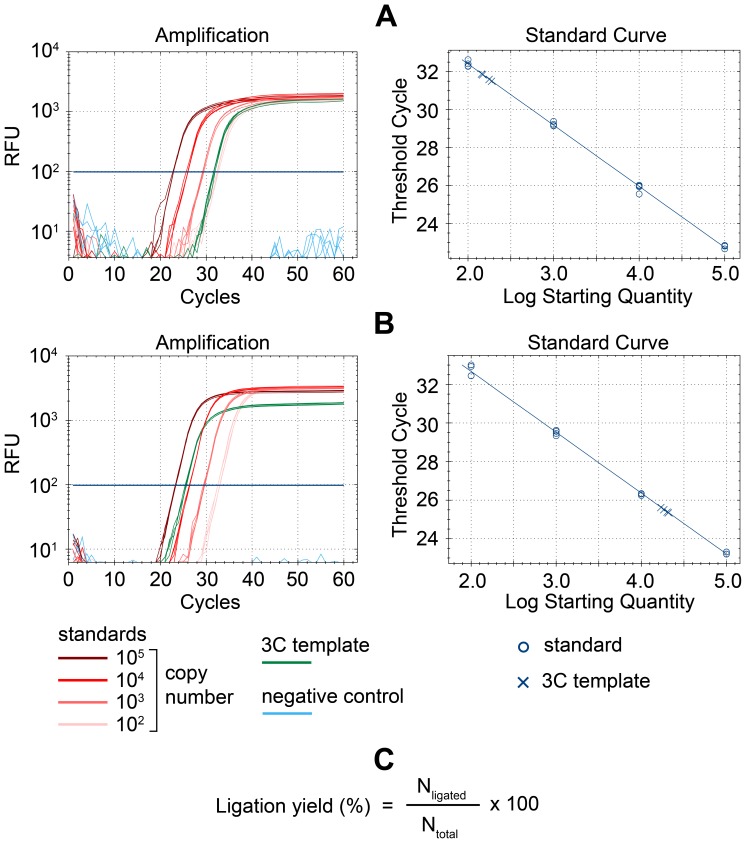
Results of amplification used to estimate the yield of the *Hbb-b1* promoter–HS4/5 ligation product. (A) Primers used to amplify the *Hbb-b1* promoter–HS4/5 ligation junction. (B) Primers to an inner portion of the HS4/5 restriction fragment (control amplification). Fluorescence growth curves (logarithmic scale) are presented on the left graphs. Green curves show the results for the 3C template; blue curves show the results for the negative control (50 ng of mouse genomic DNA (A) or mQ (B)); red curves of different color intensity show the results for the DNA standards. Standard curves are presented on the right graphs. (C) The equation used to calculate the yield of ligation products. N_total_–the quantity of each of the two restriction fragments; N_ligated_–the quantity of the ligation product of these two fragments.

This approach allowed us to determine the precise numbers of different ligation products in the 3C template. For example, Figure 2A shows the results of amplification of the *Hbb-b1* promoter–HS4/5 ligation product. The copy number of the ligation product in 50 ng of the 3C DNA sample was determined to be ∼170. This amount of the sample should contain approximately 18000 copies of each genomic fragment (based on the size of the haploid mouse genome, which is ∼2.7 billion base pairs, according to the GRCm38/mm10 assembly). Using the equation presented in Figure 2C, the actual yield of the target cross-ligated product in this case is calculated as approximately 0.9%. To ensure the accuracy of our calculations, we directly quantitated the restriction fragments of interest using qPCR with primers to the inner regions of the restriction fragments. The standard sample was prepared in the same fashion as the standard for quantification of ligation products (see above). Figure 2B shows the amplification results for the HS4/5 restriction fragment. The copy number of the restriction fragment in 50 ng of the 3C DNA sample was determined to be ∼19000, which is very close to the figure calculated based on the mouse genome size. Similar results were observed for the other restriction fragments studied (data not shown).

**Figure 3:**
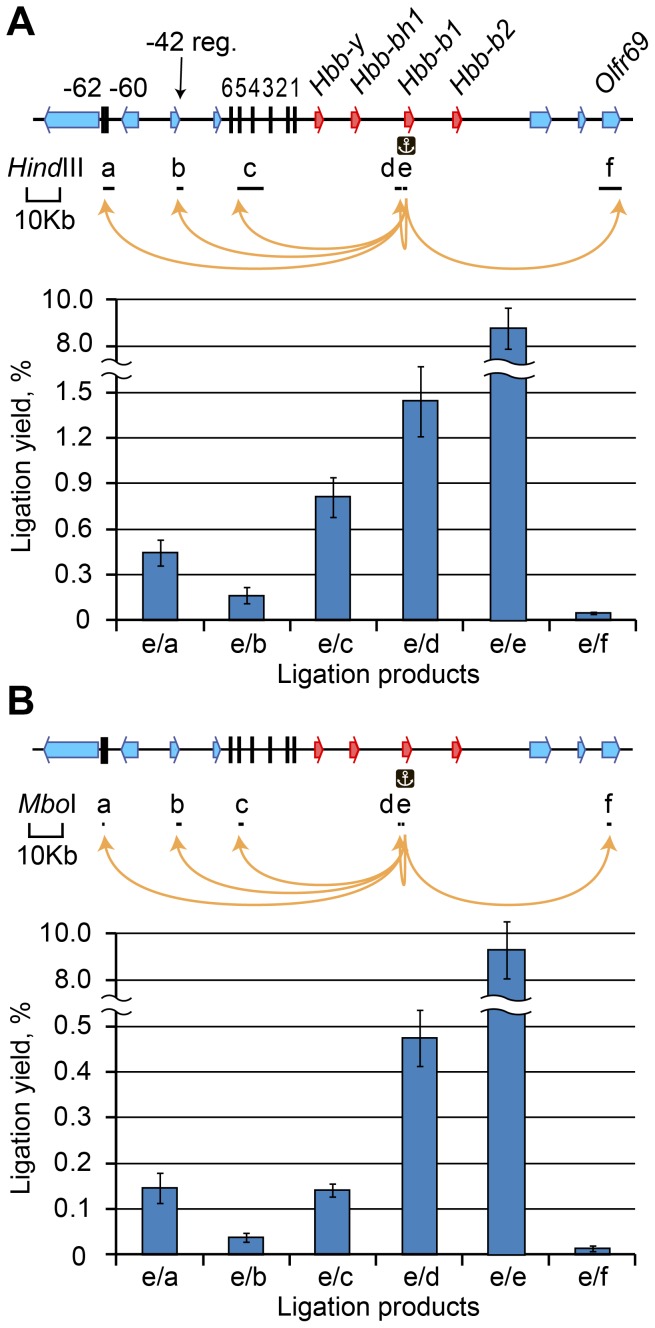
Yield of ligation products for different fragments of the mouse beta-globin gene domain. (A) The results for *Hind*III-digested fragments. (B) The results for *Mbo*I-digested fragments. Above each graph, a map of the domain is shown (beta-globin genes, red arrows; olfactory receptor genes, blue arrows; DNase I hypersensitive sites, black vertical lines). Black horizontal lines below the map show the positions and sizes of the analyzed restriction fragments. The individual fragments are designated by lowcase letters. An anchor symbol indicates the anchor restriction fragment. The ligation yield was calculated using the equation presented in Figure 2C. The ligation products are named according to the fragment designations; e/e–self-ligated product. The error bars represent the SEM of three independent experiments.

An analysis of the ligation frequencies of different fragments of the beta-globin gene locus performed using the approach described above (Figure 3A) confirmed the previously reported differences in the frequencies of ligation of the *Hbb-b1* promoter to various fragments of the locus [2]. However, the absolute frequencies of ligation were quite low in all cases. For example, the HS -62/-60, a regulatory element believed to participate in the assembly of an active chromatin hub along with the HS4/5 of the LCR, was ligated to the *Hbb-b1* promoter with a frequency of 0.4–0.5% (ligation product “e/a” in Figure 3A), while the regions assumed to be looped out from the hub, such as the -42 region and the olfactory receptor gene *Olfr69*, showed ligation frequencies of ∼0.15 and 0.05%, respectively (Figure 3A, ligation product “e/b” and “e/f”). The highest ligation frequency (approximately 1.5%) was observed for the fragment located directly upstream of the anchor fragment (Figure 3A, ligation product “e/d”). Using the above described experimental approach we also estimated the yield of the circularized (self-ligated) anchor fragment. It was found to be ∼9% (Figure 3A, ligation product “e/e”).

We next performed 3C experiments using a frequently cutting restriction enzyme (*Mbo*I) and analyzed the ligation frequencies of the same sites of the beta-globin gene locus that were analyzed in the experiments with *Hind*III enzyme. The total level of ligation was approximately three times lower in the *Mbo*I experiments than in the *Hind*III experiments (Figure 3B). In contrast, the yield of the circularized *Mbo*I bait fragment was ∼10% (Figure 3B, ligation product “e/e”), i.e. very similar to the yield of the circularized *Hind*III anchor fragment (see above).

Taking into account the relatively low frequencies of ligation observed in our experiments, we checked the completeness of the restriction enzyme digestion, which might influence the yield of ligation. The restriction enzyme digestion in the 3C procedure is carried out in the presence of 0.1–0.3% SDS; the digestion reaction uses a large excess of the enzyme to overcome the inhibitory effects of SDS and allow the reaction to proceed to completion. Moreover, Triton X-100 is added to the solution to sequester the SDS and thus help to improve the yield of the digestion reaction. However, even under these conditions many restriction enzymes do not work properly [4]–[6]. Using PCR-stop analysis, we determined the efficacy of cleavage of the beta-globin gene locus at different sites throughout the locus and found it to be >85% for *Hind*III restriction enzyme (which is in agreement with the previously published data [2], [7]–[9]) and >70% for *Mbo*I restriction enzyme (data not shown).

**Figure 4:**
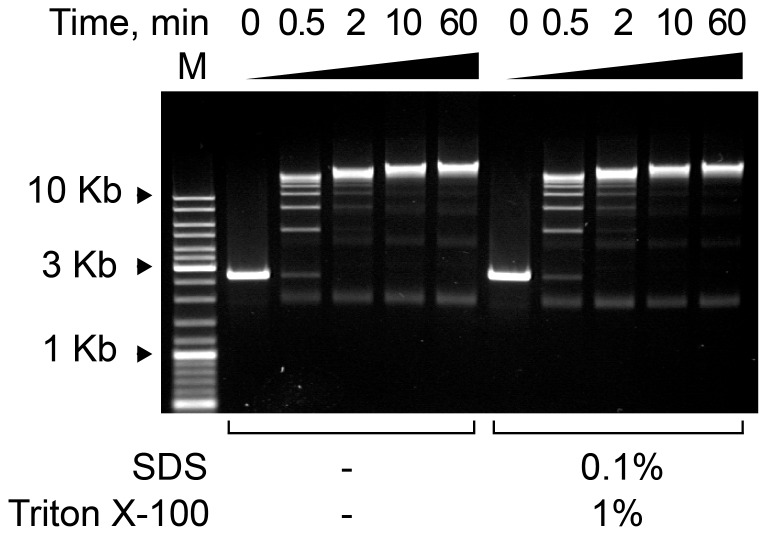
The kinetics of the ligation reaction in the presence or absence of SDS. Electrophoretic separation of the products obtained upon the ligation of pUC18 *Hind*III fragments for the indicated times in the presence or absence of SDS and Triton X-100 in an ethidium bromide-stained agarose gel. The reaction was carried out in 1× T4 DNA Ligase Buffer (Fermentas) with 100 ng/ µl DNA and 0.1 U/ µl T4 DNA ligase (Fermentas). M–DNA size marker (Fermentas, SM0331).

We also evaluated whether the ligase functions properly under the conditions used in the 3C assay. The ligation step of the 3C procedure is performed in the presence of 0.1% SDS and 1% Triton X-100. To determine whether the presence of these detergents influences the efficacy of the ligation, we compared the kinetics of ligation of the *Hind*III-fragments obtained by linearizing the pUC18 plasmid in the presence and absence of detergents. The results presented in Figure 4 demonstrate that, under the conditions used in our experiments, SDS does not interfere with the ligation reaction.

## Discussion

The proximity ligation is the key step in all C-methods [2], [3]. In this work, we have demonstrated that the actual ligation frequencies of the DNA fragments assumed to be assembled in a chromatin hub are in the range of tenths of a percent. This low frequency of ligation was observed between fragments containing the promoter of an active beta-globin gene, *Hbb-b1*, and its distant enhancers. At the same time, the frequency of ligation of “negative controls”, such as the -42 region and *Olfr69* gene, to the *Hbb-b1* promoter was below 0.1% in most cases. In our experiments, we used two restriction enzymes, either a standard 6-bp cutter (*Hind*III) or a frequent cutter (*Mbo*I). Although the characteristic differences in ligation frequencies observed for different pairs of fragments were well reproduced between the experiments with these endonucleases, the overall level of ligation in the experiment that used *Mbo*I digestion was appreciably lower.

The low yield of the 3C ligation products observed in our experiments may have different explanations. First of all, the technical reasons should be considered. It is obvious that each restriction fragment has two cohesive ends. If both ends of an anchor fragment and the interacting fragment are taken into consideration, the total yield of the ligation products should be increased about four times. In a putative active chromatin hub the anchor fragment is likely to be juxtaposed to many other restriction fragments including its own neighboring fragments and the neighboring fragments of the interacting regulatory elements. Assuming that the probability of ligation with different DNA fragments located in a close proximity to the anchor is about the same, one would expect the yield of ligation products with any particular cohesive end available for ligation to be reverse-proportional to the number of such cohesive ends. This appears to be the case in our experiments. Indeed, *Mbo*I introduces much more cuts than *Hind*III, and the yield of specific ligation products in the *Mbo*I-3C experiments was found to be significantly lower than the yield of specific ligation products in the *Hind*III-3C experiments.

On the other hand, all the above-mentioned factors should also affect the probability of self-ligation (circularization) of the anchor fragment. In a recent study [11] we have demonstrated that, in contrast to the current model [2], [3], [12], SDS extraction does not cause a lysis of formaldehyde-fixed nuclei and does not result in a solubilization of a substantial amount of cross-linked chromatin fragments. Consequently, the proximity ligation in a 3C protocol occurs within non-lysed nuclei in a chromatin cage stabilized by formaldehyde cross-links. In such a chromatin cage the probability of ligation of the ends of all restriction fragment located in a spatial proximity will likely be similar if not the same. Yet, we have found that the yield of circularized anchor fragment exceeds at least 10 times the yield of the ligation products of this fragment with any distant DNA fragment assumed to be assembled in a common active chromatin hub with the anchor fragment. Furthermore, the yield of the circularized anchor fragment was about the same in *Hind*III-3C and *Mbo*I-3C experiments. Thus, it was not affected by the presence of additional cohesive ends available for ligation. The simplest explanation for these observations is that a significant portion of the anchor fragments is not cross-linked to any other restriction fragment (including the one located just upstream to the anchor, as the yield of the cross-ligation products in this case was only 1.5% versus 10% of circularization).

The probability of cross-linking of different regions of a folded chromatin fiber may depend on various factors including the efficiency of the cross-linking reaction *per se* and the actual frequency with which the target DNA fragments interact in the nucleus. In this regard, it should be noted that most cells in the population used for this study (E14.5 fetal liver cells) are erythroid precursors that transcribe the *Hbb-b1* and *Hbb-b2* genes [13], [14]; interactions between the beta-globin genes and their enhancers might be anticipated for all these cells. In this light, the low frequencies of ligation observed in our experiments may well reflect that these interactions are not stable or uniform enough to support the cross-linking of the corresponding fragments of a chromatin fiber in the majority of cells present in the population. Several previous observations also strongly suggest that the interactions between the promoters and enhancers of beta-globin genes are short-lived and dynamic [15], [16]. Additional experiments will be necessary to obtain further insights into the nature of interactions between DNA regulatory elements.

Whatever is the reason for low levels of the ligation products in the 3C experiments, this can be a source of different artifacts. The lower is the level of the semantic signal, the higher is the possibility to disturb the message by unaccounted factors. There are many factors that can affect the efficiency of the proximity ligation. Besides the spatial proximity of the restriction fragments under study, the condition of the cohesive ends (e.g., their lengths and mobility, the presence of cross-linked proteins, etc.) should determine their ability to reach each other and be ligated. Taking into account the fact that, even in experiments with such a classical model system as the mouse beta-globin gene domain, the levels of ligation products in the maximums and the minimums of the 3C curves do not exceed 1.5%, one cannot not ignore the possibility that the profiles of 3C curves may be affected by the factors other than the spatial proximity. The necessity of various controls in the 3C experiments has been intensively discussed in the literature [1], [17], [18]. The results presented in this paper further reinforce the importance of this issue.

## Materials and Methods

### Mice

All experiments were approved by the Animal Care and Use Committee of the Institute of Gene Biology of the Russian Academy of Sciences. All mice were provided with nesting material and housed in cages maintained under a constant 12-h light/dark cycle at 21 to 23°C, with free access to standard chow and tap water.

### 3C analysis

3C was performed as previously described [2], [4]. Briefly, pregnant mice were sacrificed by cervical dislocation at E14.5 and embryos were dissected from the uterus. After surgical removal, the fetal livers were disrupted by pipetting in DMEM medium supplemented with 10% fetal bovine serum (FBS) and passed through a 40 µm cell strainer to produce a single-cell suspension. An aliquot containing 10^7^ cells was treated with 2% formaldehyde in PBS/10% FBS for 10 min at room temperature, and the reaction was stopped by adding glycine to a final concentration of 0.125 M. After washing with PBS/10% FBS, the fixed cells were incubated for 10 min in an ice-cold lysis solution (10 mM Tris pH 8.0, 10 mM NaCl, 0.2% Nonidet P40, and protease inhibitor cocktail (Fermentas)) at a concentration of 2×10^7^ cells/ml to release the nuclei. The nuclei were harvested and suspended in 0.5 ml of 1.2× restriction buffer 2 (New England Biolabs) for subsequent *Hind*III digestion or 0.25 ml of 1.2× restriction buffer 3 (New England Biolabs) for *Mbo*I digestion. SDS was added to a final concentration of 0.3%, and the solution was incubated for 1 h at 37°C with shaking. In the case of *Mbo*I digestion, the incubation was followed by the addition of 0.25 ml of 1.2× restriction buffer 3. Triton X-100 was added to 1.8%, and the solution was further incubated for 1 h at 37°C to sequester the SDS. The DNA was digested by overnight incubation with 600 units of *Hind*III or 800 units of *Mbo*I (New England Biolabs) at 37°C with shaking. The restriction endonuclease was inactivated by adding SDS to a final concentration of 1.6% and incubating for 20 min at 65°C. The solution was diluted by adding 7 ml of 1× ligation buffer (Fermentas). Triton X-100 was added to 1%, and the solution was incubated at 37°C for 1 h with shaking. Next, 100 U of T4 DNA Ligase (Fermentas) was added, and the DNA was ligated for 4.5 h at 16°C and then for 30 min at room temperature with slow agitation. Cross-links were reversed by incubation at 65°C for 16 h in the presence of Proteinase K (40 µg/ml). After cross-link reversion, RNase A was added to a final concentration of 40 µg/ml, and the RNA was digested for 45 min at 37°C. The DNA was purified by extraction with phenol, phenol-chloroform and chloroform, followed by precipitation with ethanol. For the subsequent analysis of anchor fragment circularization, the 3C samples were treated with a restriction enzyme cutting inside the anchor fragment outside of the amplification region, followed by DNA purification. The DNA concentration was determined using a fluorometric assay (Qubit, Invitrogen).

The ligation products were analyzed by TaqMan real-time PCR. The 20 µl real-time PCR reaction contained 50 ng of the 3C DNA template or the same amount of the mouse genomic DNA along with required quantity of a standard DNA, 1× PCR buffer (50 mM Tris (pH 8.6), 50 mM KCl, 1.5 mM MgCl_2_, and 0.1% Tween 20), 10 pmol of each primer, 5 pmol of a TaqMan probe (5′-FAM dye, inside BHQ-1 quencher), 4 nmol of each dNTP and 1 U of Hot start Taq DNA polymerase (SibEnzyme). The reaction was performed in a BioRad CFX96 PCR machine as follows: initial denaturation for 5 min at 94°C, then 60 cycles of 15 s at 94°C and 60 s at 60°C, followed by a reading of the plate at the end of each cycle. The sequences of the primers and TaqMan probes used are presented in Table 1.

**Table 1:** Sequences of the primers and TaqMan probes used for 3C analysis.

Fragment	Test region	Primer/TaqMan set (5′-3′)
a-*Hind*III	HS-62/-60	a/s GGGTGTGGGTATTTGTAAGAG
b-*Hind*III	-42 region	a/s ATGAACAAGTTTCATGGGG
c-*Hind*III	HS4/5	a/s TTCAAGTTCTCATCCTTCACTG
d-*Hind*III	upstream of *Hbb-b1* promoter	s AGAAGGAGATTCATCCATGCACT
e-*Hind*III	*Hbb-b1* promoter (anchor)	s AATCGCTGCTCCCCCTCACTTM FAM-ACCAAAGAAAGAGGAAA(T-BHQ1)GACAACACAGAACA-PO_4_
e-*Hind*III	*Hbb-b1* promoter (opposite end)	a/s CAACACATTTGCTCAATCAACTACT
f-*Hind*III	*Olfr69*	s ACTGCACTGTCTTCCAAATCACT
a-*Mbo*I	HS-62/-60	s TGTAGTTCTCTAGTGTAGCCACCAG
b-*Mbo*I	-42 region	a/s TAGATGCATGGTCTTAATGGTCC
c-*Mbo*I	HS4/5	a/s TACTAATAAAAGCAAGCCATCTCG
d-*Mbo*I	upstream of *Hbb-b1* promoter	s TGAGGACTTGGTTCAGTAAATAA
e-*Mbo*I	*Hbb-b1* promoter (anchor)	s CTGATTCCGTAGAGCCACACCTM FAM-CCTACCTCACC(T-BHQ1)TATATGCTCTGCCCTG-PO_4_
e-*Mbo*I	*Hbb-b1* promoter (opposite end)	a/s ACTGCCTTCAGAGAATCACCCT
f-*Mbo*I	*Olfr69*	s AAAACAAGATGAGAATCGCCTG

s, sense primers; a/s, antisense primers (with respect to the direction of transcription of beta-globin genes); TM, TaqMan probes.

For the analysis of restriction fragment quantities (PCR with internal amplicons), the mouse genomic DNA was not added to the DNA standards. The sequences of the primers and TaqMan probe used for analysis of HS4/5 fragment are as follows: 5′-TACAAGTCTCCTCATGTTCCCAA-3′, 5′-TTTTCAGACATACTCCTCTATCCAG-3′, and 5′-FAM-TAGCCTCAGT(T-BHQ1)ACCCGACATTAGTTCTTAGT-PO_4_-3′.

### Random ligation mix preparation

A BAC random ligation template was generated using a bacterial artificial chromosome carrying the murine beta-globin gene locus, along with flanking sequences (BAC clone RP24-79I7, CHORI BACPAC Resources Center), that had been digested with *Hind*III or *Sau*3A (an isoschizomer of *Mbo*I that is insensitive to dam methylation) and then ligated at a high DNA concentration. The obtained template was amplified with the target 3C primers (see Table 1). The amplification products were separated on an agarose gel and purified using a QIAGEN gel extraction kit. The purified products were subjected to a fluorometric assay (Qubit, Invitrogen) to determine their DNA concentration and subsequently mixed in equimolar amounts.
